# The Relationships between Abnormal Serum Lipid Levels, Depression, and Suicidal Ideation According to Sex

**DOI:** 10.3390/jcm11082119

**Published:** 2022-04-11

**Authors:** Kounseok Lee, Sunhae Kim, Jung Ki Jo

**Affiliations:** 1 Department of Psychiatry, Hanyang University Medical Center, Seoul 04763, Korea; dual@hanyang.ac.kr (K.L.); sunhk0906@hanyang.ac.kr (S.K.); 2 Department of Urology, Hanyang University Medical Center, Seoul 04763, Korea

**Keywords:** cholesterol, high-density lipoprotein, triglycerides, depression, suicidality, sex

## Abstract

(1) Background: Serum lipid levels affect not only nutritional status but also emotional state. The purpose of this study was to examine the effects of various socio-demographic characteristics, abnormal cholesterol levels, and BMI indicators on depressive symptoms and suicidal ideation in the Korean population. (2) Methods: A total of 23,692 people were surveyed using data from the Korea National Health and Nutrition Examination Survey (KNHNES) 2014, 2016, and 2018. Data from 11,653 patients were analyzed. Age, sex, chronic disease, smoking, alcohol consumption, total cholesterol (HDL, triglycerides), BMI, depression, and suicidal ideation were measured. (3) Results: According to sex, low HDL, high triglycerides, and suicidal ideation were significant, along with low education level, smoking, binge drinking, and high BMI. High triglyceride level was shown to significantly increase the risk of depression in males (OR = 1.535, 95% CI = 1.098–2.147). Factors affecting suicidal ideation in males were age, binge drinking, and depression, while blood lipid factors were not significant. (4) Conclusions: Of the types of serum lipid factors affecting depression and suicidal ideation, high triglycerides were found to be a risk factor for depression in men. Serum lipids can be used as biomarkers to reflect depressive symptoms in men depending on cholesterol level.

## 1. Introduction

Serum lipids reflect an individual’s constitution and nutritional status and are significantly affected by emotional status [1]. In particular, cholesterol is a key member of the central nervous system (CNS) and is essential for the stability of cell membranes and proper nerve transmission [2]. Cholesterol is also related to the mechanism of action of antidepressants and mood stabilizers and plays an important role as a secondary messenger in the brain system involved in mood disorders [3,4].

According to the stress–diathesis model, suicide is associated with environmental stress; in particular, environmental stress in people with vulnerable constitutional predispositions such as genetic factors, family history, neurobiological characteristics, developmental factors, and personality characteristics. Suicide also is affected by substance abuse, mental illness, and/or physical illness [5]. Among the neurobiological factors related to suicide and genes, neurotransmitters and hormones can induce suicide-related mental disorders or increase susceptibility to suicide independent of mental disorders [6]. Cholesterol levels can be increased due to occupational stress or acute mental stress, and depending can affect the brain system and constitutional factors such as depression and suicide risk factors.

Interest in cholesterol and suicide has increased as a result of cholesterol treatment to lower the risk of cardiovascular disease and the associated increasing non-illness-related mortality [7,8,9]. In addition, since most causes of death in follow-up studies of low cholesterol levels and non-disease-related deaths were suicide [10,11,12], studies on the relationship between low cholesterol levels [13,14,15] and suicide attempts have continued [16,17].

Based on these studies, the relationship between suicide and depression according to cholesterol type and the level was elucidated. Low total cholesterol is associated with suicide [18], and patients with major depression have lower total cholesterol than those without depression [19,20]. Furthermore, there was a correlation between serum cholesterol and suicide attempts in major depressive disorder patients (md = −19.9 mg/dL, *p* = 0.004), so both factors are major in serum cholesterol [21]. In addition, patients with depression have lower concentrations of high-density lipoprotein cholesterol (HDL) and high ratios of total cholesterol/high-density lipoprotein cholesterol (TC/HDL) and low-density lipoprotein cholesterol/high-density lipoprotein cholesterol (LDL/HDL) [12,22,23]. In previous studies, severe depressive symptoms occurred as a result of decreased cholesterol levels by treatment with pravastatin in hypercholesterolemic patients [24], associated with lowering of serum TC and LDL cholesterol. In males, it was found to be associated with depressive symptoms and low levels of HDL cholesterol [8,9,12].

As such, many studies have found that cholesterol is associated with suicide rates and depression [10,25] and that people with very low blood cholesterol are at a higher risk of suicide and a higher actual suicide rate [11,15,26]. These results indicate that blood cholesterol reflects the cholesterol content of the brain, and a decrease in lipids in the neuronal cell membrane causes 5-hydroxytryptamine (5-HT) receptor dysfunction and decreased neural synapse formation [26]. In the frontal lobe of suicide attempters, lipid metabolism-related fatty acid desaturase (FADS1), leptin receptor (LEPR), phosphoinositide-3-kinase class 2 alpha (PIK3C2A), and stearoyl-CoA desaturase (SCD) are downregulated [27].

Although the neurobiological principles of the effects of serum lipids and cholesterol on suicide and depression were studied, cholesterol levels are inconsistent and not integrated. Contrary to studies showing a relationship between low cholesterol levels and depressive symptoms, some studies have indicated higher serum cholesterol in the suicidal ideation group [28], no relationship between low serum cholesterol and depressive symptoms [24], or an inverse correlation [29]. Moreover, serum cholesterol levels in some depressed patients were reported to be within the normal range [30]. Although cholesterol levels for suicide risk have been shown to differ based on sex (202 mg/dL for men and 205 mg/dL for women) [31], or in other studies of the correlation between serum cholesterol and suicide attempt in MDD (major depressive disorder), latent variables excluding men did not play any role in the correlation [21], so additional research is needed.

Although studies on the relationships between cholesterol and depression, suicide, and aggressive behavior are progressing, consistent conclusions cannot be drawn, and the relationship between cholesterol and sex is lacking. Therefore, this study examined whether cholesterol type is related to suicide and depressive symptoms according to sex using the results of the Korea National Health and Nutrition Examination Survey (KNHNES), a large-scale survey conducted by the Korea Centers for Disease Control and Prevention.

## 2. Materials and Methods

### 2.1. Participants

The National Health and Nutrition Survey is a nationwide survey conducted based on Article 16 of the National Health Promotion Act enacted in 1995 in Korea. National health level, health behavior, and food and nutritional intake are representative and reliable statistics. The basics of health policy, such as setting and evaluating goals of the comprehensive national health promotion plan and developing health promotion programs, are designed using these data. In this study, data from the Korea National Health and Nutrition Examination Survey (KNHNES) from 2014, 2016, and 2018 contain items on depression. From a total of 23,692 people surveyed, 7064 who did not provide an answer on depression were excluded. Among the remaining people, 2914 did not respond to suicidal items and were excluded. Finally, data from a total of 11,653 participants were analyzed. This study was approved by the Research Ethics Review Committee of the Korea Centers for Disease Control and Prevention (2018-01-03-P-A).

### 2.2. Measurements of General Variables

Measured variables were age, sex, chronic disease, smoking, alcohol consumption, total cholesterol (HDL, triglycerides), BMI, depression, and suicidal ideation. Chronic diseases comprised heart attack, osteoarthritis, diabetes, thyroid disease, hypertension, dyslipidemia, cerebral infarction, and pulmonary tuberculosis. Income was divided into quartiles. A low level of education was defined as no education to graduation from middle school. Patients who currently smoked were defined as smokers, and those who consumed alcohol more than twice a week were defined as heavy drinkers. Suicidal ideation was noted in the presence of suicidal thoughts or plans in the past year. The BMI was calculated using measured weight and height and was classified into three groups according to the World Health Organization standards: less than 18.5 m^2^/kg, underweight; 18.5 to 24 m^2^/kg, normal weight; and over 25 m^2^/kg, overweight.

#### 2.2.1. Measurement of Lipid Levels

Blood samples from individual participants were collected for the determination of total cholesterol, high-density lipoprotein cholesterol (HDL-C), and triglycerides. Interlaboratory comparisons using the same samples were performed according to the guidelines of CLSI EP9-A2-IR (Clinical and Laboratory Standards Institute, PA, USA). Whole blood samples (5%) were re-evaluated to determine the reproducibility of test results. Since 2009, KNHNES has measured HDL-C levels according to the Centers for Disease Control and Prevention (CDC) Lipid Standardization Program [32]. Blood sample levels were defined as abnormal when total cholesterol was 200 or more, triglycerides were 150 or more, and HDL was 60 or less.

#### 2.2.2. Measurement of Depression

The Patient Health Questionnaire (PHQ) is a self-report questionnaire designed to help detect and diagnose mental disorders that are common in primary clinical settings [33]. The questionnaire consists of nine items rated based on frequency over the last two weeks. In this study, the cut-off point was set to 10, and any score higher than that was defined as depression.

### 2.3. Statistical Analysis

For general characteristics of the subjects, frequency and cross-analyses (χ^2^-test) were performed to confirm the differences in variables according to sex. Logistic regression analysis was performed to determine the effects of abnormal clinical characteristics and lipid indicators on suicidal ideation or depression, and the odds ratio (OR) and 95% confidence interval (95% CI) were calculated. In order to assess the multicollinearity between variables, the tolerance limit and variance inflation factor (VIF) was calculated. All statistical analyses were performed using IBM SPSS statistics (version 24.0, IBM Corporation, New York, NY, USA), and the statistical significance level was set to 0.05.

## 3. Results

### 3.1. Experimental Results

#### General and Clinical Characteristics

Of the total 11,653 participants, 5049 were male (43.3%), and the average age was 51.07 years (SD = 16.77). Average total cholesterol was 192.12 mg/dL (SD = 37.65), and that of HDL cholesterol was 51.15 mg/dL (SD = 12.81, Table 1). As for the clinical characteristics according to sex, low HDL, high triglycerides, and suicidal ideation were significant, along with low education level, smoking, drinking, and BMI. Regarding abnormal serum lipid levels, a larger percentage of males than females had low HDL-C (87.4%) and high triglycerides (39.0%), indicating a significant sex difference (*p* < 0.01, Table 2).

### 3.2. Serum Cholesterol and Depression

Regression analysis confirmed that the VIF value was less than 10 in all models, and there was no multicollinearity. Of the clinical factors affecting depression, low-income level and low education level were related regardless of sex, as was smoking (Table 3). The largest clinical factor for depression in females was smoking (OR = 4.091, 95% CI = 3.059–5.472). High triglyceride levels had a significant effect on depression only in males (OR = 1.535, 95% CI = 1.098–2.147, Table 3).

### 3.3. Serum Cholesterol and Suicidal Ideation

As for risk factors affecting suicidal ideation, clinical factors were more significant than abnormal serum lipid factors (Table 4). In terms of clinical factors, age, in particular, had an effect on male suicidal ideation, and only in males did the risk increase as the age group increased. The highest level of suicidal ideation was shown in the elderly group over 64 years of age (OR = 3.430, 95% CI = 1.123–3.144, Table 4). Low-income level significantly increased suicidal ideation in both males and females (male OR = 1.879, 95% CI = 1.123–3.144; female OR = 1.624, 95% CI = 1.074–2.455), and low education level was significant only in females. Drinking significantly increased suicidal ideation in females (OR = 1.669, 95% CI = 1.16–2.519). As previously known, depression was the biggest risk factor for suicidal ideation in both males and females. Among all clinical factors and serum lipid factors, depression in males significantly on suicidal ideation (male OR = 10.794, 95% CI = 6.053–19.248; female OR = 7.526, 95% CI = 4.904–11.549, Table 4). No serum lipid factors (total cholesterol, HDL-C, TG) were significant. In both suicidal ideation and depression, neither BMI nor chronic disease was a significant clinical factor (Table 3 and Table 4).

## 4. Discussion

In this study, abnormal serum lipid factors affecting suicide and depression were analyzed along with clinical factors. Unlike previous studies, cholesterol, HDL cholesterol, and TG were analyzed as serum lipid factors affecting depression and suicidal thoughts. High TG significantly increased in the depression phase only in males (OR = 1.535, 95% CI = 1.098–2.147). As in previous studies, the strongest predictors of suicidal ideation were depression and male age. As for demographic factors, smoking was significantly associated with depression, and drinking was significantly associated with suicidal thoughts in both males and females.

Data from the National Health and Nutrition Survey in the United States found that low HDL-C was associated with an increased prevalence of suicide attempts in females, but no significant association was found in males [19,34]. Among patients with major depressive disorder, patients with recent suicide attempts had lower serum TG levels and higher HDL-C levels than healthy controls [9,35], and suicide attempters with mood disorders had lower TG levels than healthy controls [36]. However, a recent study examining the relationship between serum cholesterol levels and depressive symptoms in a Korean sample found that the higher was the HDL-C level in middle-aged adults [37], the higher was the risk of depression, and the lower blood cholesterol level in the elderly aged 60 years or older [38]. There was also a study that found no association between suicidal ideation and suicide attempts. The cause of these different results might be the difference in diet between Western and Korean societies. Moreover, in the case of triglycerides, one study shows no certain pattern of TG during stress [39].

The associations between high TG levels and depression and suicide, which were significant in this study, can be explained by a psycho-neuroimmunological hypothesis where a T-cell-mediated immune reaction is activated in depressed patients [40]. As a result, the production of cytokines, including interleukin-2 (IL-2), is increased, which lowers the concentration of serum cholesterol, especially HDL, and increases serum TG to inhibit the secretion of melatonin from the pineal gland. This induces an increase in impulsivity and suicide. Therefore, the significance of high TG in depression supports the epidemiological hypothesis related to serum cholesterol concentration. In particular, serum TG was significantly linked to depressive symptoms, indicating that they affect psychological characteristics [28,41,42,43].

Previous studies showed a correlation between low HDL levels and depressive symptoms, but a Korean study showed no such correlation [44]. Although HDL is a protective factor for coronary artery disease, it is thought to show more complex relationships with depression and suicide. Although many studies showed an association between serum lipid levels and depression and suicide, most compared serum TC levels and comparative studies between serum lipids are lacking. In addition, conflicting results were reported on the relationships between serum lipid levels and suicide; serum cholesterol and TG levels were not lower in survivors of suicide attempts [45], and the association between low serum cholesterol and suicide attempts was not confirmed by international replication studies [46].

This study has strengths as epidemiological research uses a relatively large sample size to reduce the overall discrepancy between cholesterol, suicide, and depression. We found that TG is the most significant factor affecting depression in males and plan to investigate whether the mechanism of change in serum lipid concentration due to stress can mediate depression. In addition, international replication studies of serum cholesterol and suicide and depression were verified, and the effects of serum cholesterol on psychiatric factors were supported. Moreover, of studies analyzing serum cholesterol, depression, and suicide, ours has the strength of analyzing serum lipid factors (TC, HDL cholesterol, TG) and depression and suicidal ideation separately. This study identified an overall relationship between depression and serum lipid factors on suicide by integrating clinical factors. Though serum TG level is closely related to BMI [47], this study showed no association between BMI and either suicidal ideation or depression. Relationships between clinical factors should be verified.

Despite the strengths of this study, its limitations are as follows. First, causal relationships between serum lipid level and depression and suicidal ideation could not be identified due to the cross-sectional nature of the study. Second, suicide is a measure of suicidal ideation, and more severe suicidal tendencies such as suicide attempts and suicidal behaviors were not analyzed. Third, for TG less than 200 mg/dL, the LDL cholesterol level was not disclosed in the raw data, preventing analysis of the effect on LDL cholesterol. Fourth, medical conditions thought to be related to depression or drug history that could affect serum lipid concentrations were not taken into account.

The relationships between serum lipids and depression and suicide remain controversial. Meanwhile, omega-3 fatty acids are thought to be helpful in the prevention and treatment of various mental disorders due to their antidepressant effects [48]; research on the relationships between lipids and depression and suicide has received renewed attention [49]. The results of this study partially explain why omega-3 fatty acids, such as ∂-linolenic acid, have an effect on depression.

## 5. Conclusions

Serum lipid levels can be used as biomarkers to reflect the risk of depression and suicide. This study found that high TG levels among TC, HDL cholesterol, and TG significantly increased the risk of depression in male subjects.

## Figures and Tables

**Table 1:** General characteristics of the participants (*n* = 11,653).

Variable	Value
age (years)	51.07 ± 16.77
sex (male)	5049 (43.3%)
chronic disease	2256 (19.4%)
smoker	2136 (18.3%)
drinker	3851 (33.3%)
TC	192.12 ± 37.65
HDL	51.15 ± 12.81
TG	137.10 ± 115.19
BMI	23.98 ± 3.55
PHQ-9 total	2.52 ± 3.66

Values are presented as mean ± SD or *n* (%). TC: total cholesterol; HDL: the high-density lipoprotein cholesterol; TG: triglyceride; BMI: body mass index; PHQ-9: Patient Health Questionnaire-9.

**Table 2:** Clinical characteristics according to sex.

		Male (*n* = 5030)		Female (*n* = 6573)		χ^2^	*p*
		*n*	%	*n*	%		
age (years)	19–44	1940	38.4%	2445	37.0%	3.741	0.15
	44–64	1835	36.3%	2511	38.0%		
	≥64	1274	25.2%	1648	25.0%		
income (low)		1240	24.6%	1607	24.3%	0.079	0.79
education (low)		1071	21.2%	2204	33.4%	209.45	<0.01
chronic diseases		1029	20.4%	1227	18.6%	5.942	0.02
Smoker		1761	34.9%	375	5.7%	1629.71	<0.01
Drinker		2005	39.7%	1846	28.0%	178.79	<0.01
TC (high)		1891	38.3%	2547	40.1%	3.551	0.06
HDL (low)		4311	87.4%	4408	69.4%	512.932	<0.01
TG (high)		1924	39.0%	1382	21.7%	399.215	<0.01
BMI	<18.5	114	2.3%	313	4.8%	178.774	<0.01
	18.5–25	2847	56.6%	4275	65.0%		
	≥25	2069	41.1%	1985	30.2%		
suicidal ideation (yes)		77	1.5%	113	1.7%	54.211	<0.01
depression (yes)		4864	96.3%	6156	93.2%	0.617	0.43

Data shown are *n* (participant numbers) and percentage. Calculated by cross-analysis (χ^2^-test). TC: total cholesterol; HDL: the high-density lipoprotein cholesterol; TG: triglyceride; BMI: body mass index.

**Table 3:** Association between serum cholesterol and depression according to sex.

		Male	Female
age (years)	19–44	reference	reference
	44–64	0.696 (0.467–1.038)	0.768 (0.579–1.020)
	≥64	1.168 (0.722–1.890)	1.051 (0.735–1.505)
income (low)		3.271 (2.398–4.462)	1.862 (1.509–2.299)
education (low)		1.695 (1.122–2.559)	1.906 (1.426–2.546)
chronic diseases		0.760 (0.502–1.151)	0.928 (0.710–1.213)
smoker		1.850 (1.336–2.562)	4.091 (3.059–5.472)
drinker		1.187 (0.864–1.629)	1.078 (0.865–1.344)
TC (high)		0.813 (0.581–1.138)	1.010 (0.814–1.252)
HDL (low)		0.812 (0.505–1.306)	0.903 (0.710–1.149)
TG (high)		1.535 (1.098–2.147)	1.161 (0.907–1.485)
BMI	<18.5	reference	reference
	18.5–25	0.727 (0.322–1.641)	0.691 (0.439–1.086)
	≥25	0.692 (0.300–1.597)	0.822 (0.511–1.325)

Calculated by adjusted odds ratios (ORs) and 95% confidence intervals (CIs). TC: total cholesterol; HDL: the high-density lipoprotein cholesterol; TG: triglyceride; BMI: body mass index.

**Table 4:** Association between serum cholesterol and suicidal ideation according to sex.

		Male	Female
age (years)	19–44	reference	reference
	44–64	2.121 (1.047–4.296)	1.030 (0.570–1.860)
	≥64	3.430 (1.537–7.658)	0.683 (0.333–1.404)
income (low)		1.879 (1.123–3.144)	1.624 (1.074–2.455)
education (low)		1.204 (0.673–2.155)	3.013 (1.738–5.222)
chronic diseases		1.661 (0.976–2.828)	0.766 (0.453–1.297)
smoker		1.514 (0.889–2.578)	1.709 (0.926–3.153)
drinker		1.325 (0.806–2.180)	1.669 (1.106–2.519)
TC (high)		1.205 (0.710–2.045)	1.221 (0.805–1.852)
HDL (low)		0.805 (0.394–1.648)	1.303 (0.792–2.145)
TG (high)		0.656 (0.378–1.137)	0.779 (0.478–1.272)
BMI	<18.5	(-)	(-)
	18.5–25	1.153 (0.259–5.123)	1.987 (0.473–8.341)
	≥25	1.359 (0.297–6.215)	1.813 (0.418–7.854)
depression		10.794 (6.053–19.248)	7.526 (4.904–11.549)

Calculated by adjusted odds ratios (ORs) and 95% confidence intervals (CIs). TC: total cholesterol; HDL: the high-density lipoprotein cholesterol; TG: triglyceride; BMI: body mass index.

## Data Availability

The data presented in this study are available on request from the corresponding author. The data are not publicly available due to ethical restrictions.
